# Fabrication of Robust and Effective Oil/Water Separating Superhydrophobic Textile Coatings

**DOI:** 10.3390/membranes13040401

**Published:** 2023-03-31

**Authors:** Li-Heng Kao, Wei-Chen Lin, Chao-Wei Huang, Ping-Szu Tsai

**Affiliations:** 1 Department of Chemical and Materials Engineering, National Kaohsiung University of Science and Technology, Kaohsiung 807, Taiwan; 2 Department of Engineering Science, National Cheng Kung University, Tainan 701, Taiwan

**Keywords:** superhydrophobic, oil/water separation, self-cleaning, ethylenediaminetetraacetic acid, poly(dimethylsiloxane), fluorinated SiO_2_

## Abstract

A superhydrophobic (SH) surface is typically constructed by combining a low-surface-energy substance and a high-roughness microstructure. Although these surfaces have attracted considerable attention for their potential applications in oil/water separation, self-cleaning, and anti-icing devices, fabricating an environmentally friendly superhydrophobic surface that is durable, highly transparent, and mechanically robust is still challenging. Herein, we report a facile painting method to fabricate a new micro/nanostructure containing ethylenediaminetetraacetic acid/poly(dimethylsiloxane)/fluorinated SiO_2_ (EDTA/PDMS/F-SiO_2_) coatings on the surface of a textile with two different sizes of SiO_2_ particles, which have high transmittance (>90%) and mechanical robustness. The different-sized SiO_2_ particles were employed to construct the rough micro/nanostructure, fluorinated alkyl silanes were employed as low-surface-energy materials, PDMS was used for its heat-durability and wear resistance, and ETDA was used to strengthen the adhesion between the coating and textile. The obtained surfaces showed excellent water repellency, with a water contact angle (WCA) greater than 175° and a sliding angle (SA) of 4°. Furthermore, the coating retained excellent durability and remarkable superhydrophobicity for oil/water separation, abrasion resistance, ultraviolet (UV) light irradiation stability, chemical stability, self-cleaning, and antifouling under various harsh environments.

## 1. Introduction

In recent years, superhydrophobic (SH) surfaces, exhibiting a water contact angle (WCA) greater than 150° and a sliding angle (SA) lower than 10° [1,2,3,4], have attracted considerable interest in both academic research and industrial applications. They show bio-mimicking, anti-sticking, self-cleaning, water-repellence, and contamination-prevention properties [5,6,7]. Their emerging applications include oil/water separation, dust-proofing, anti-icing, and anti-bioadhesion [8,9,10,11,12,13,14]. The SH surface technique is an advanced method, which binds a bionic micro/nano rough structure with low surface free energy on the substrate to have special surface features. Previous studies have shown that the SH surface with micro/nanostructures can effectively trap a large fraction of air to prevent contact with the corrosive medium [15]. The particles of two or more scales can provide dual-scale micro/nano roughness, which is essential to achieve superhydrophobicity [16,17]. However, the major limitations in existing techniques for SH fabric treatment are their extremely low abrasion durability, chemical stability, wear resistance, and antifouling under various harsh environmental conditions [4]. Numerous efforts have been made to fabricate hydrophobic coatings with both superhydrophobicity and durability through strategies such as the fabrication of highly transparent superhydrophobic coatings via adopted 3-aminopropytriethoxysilane (APTS)-modified hollow silica nanoparticles, followed by thermal annealing and chemical vapor deposition with 1H,1H,2H,2H-perfluorooctyltrimethoxysilane [18]; fabrication of a durable polydimethylsiloxane/stearic acid/silica superhydrophobic fabric [19]; fabrication of water-repellent polyester textile using stearic-acid-modified CaCO_3_ [20]; preparation of micro-nanostructure polydimethylsiloxane/silicon dioxide composite coatings [21,22,23]; and others.

However, the property retainability of all these materials was short-term [21], particularly hydrophobic materials which have low surface energy and rough surface. Moreover, the durability of the artificial SH coatings faced enormous challenges when exposed to conditions of oil/water separation, abrasion, ultraviolet (UV) light irradiation, different pH, self-cleaning, and antifouling under various harsh conditions. Generally, the durability of the SH surface prolongs its superhydrophobicity in two ways: redepositing the low-surface-energy materials or strengthening the binding ability between the rough surface with low surface energy and the substrate. Redepositing the low-surface-energy materials is a common method; however, it is also inconvenient and expensive. Therefore, strengthening the binding ability between the rough surface with low surface energy and the substrate is a relatively convenient and cost-effective strategy.

Herein, we present an artificial way to fabricate durable superamphiphobic coatings by optimizing the three key surface parameters, namely roughness, low surface energy, and binding ability. We employed two different-sized SiO_2_ particles for constructing the rough micro/nanostructure and fluorinated alkyl silane (FAS) as low-surface-energy materials. Polydimethylsiloxane (PDMS) is a hydrophobic elastic silicone rubber, and it was used as a lubricant owing to its wear resistance, outstanding water repellence, and strong adherence [24]. Ethylenediaminetetraacetic acid (EDTA), with four carboxylic acid groups, provides high reactivity to crosslink with the substrate, and it was used to strengthen adhesion between the coating and substrate [25]. The coatings showed excellent water repellency with a WCA greater than 175° and an SA of 4°. Furthermore, the coating could retain excellent durability and remarkable superhydrophobicity for oil/water separation, abrasion resistance, UV light irradiation stability, chemical stability, self-cleaning, and antifouling under various harsh conditions.

## 2. Materials and Methods

### 2.1. Materials

In this work, all the analytical grade chemicals and reagents were used as received. Tridecafluorooctyl triethoxysilane (FAS, Dynasylan^®^ F 8261) and different sizes of SiO_2_ particles (average diameter of 12 nm and diameter of 10.5 µm) were supplied by Evonik Operations GmbH (Essen, Germany). Polydimethylsiloxane (PDMS, Sylgard^®^184) and curing agent were purchased from Dow Corning (Midland, MI, USA). Ethanol (EtOH, 99.5%) and ethylenediaminetetraacetic acid (EDTA, 99%) were obtained from ACROS^®^ Organics (Morris Plains, NJ, USA).

### 2.2. Fabrication of Superhydrophobic EDTA/PDMS/F-SiO_2_ (EPS) Coating

In a typical synthesis, 0.5 g of FAS was dissolved in 99.5 g ethanol and magnetically stirred at room temperature for 2 h. In total, 1 g of SiO_2_ particles with different sizes (12 nm: 10.5 µm = 1-9:9-1 in weight ratio) was incorporated into the prepared solution, and the mixture suspension was then dispersed by ultrasonication and mechanical stirring for 2 h to avoid the aggregation of SiO_2_ particles. Subsequently, 1 g of PDMS was added into the solution and stirred for 1 h. It was followed by the addition of 0.1 g of curing agent and 1 g of EDTA to form EPS painting. By changing the incorporating mass ratio of the different-sized particles, the samples with different proportions of SiO_2_ particles can be obtained. Finally, the dip-coating method was used to coat the prepared EPS paint on various substrates, such as glass, wood, plastic, metal, textile, and sponge. 

**Figure 1:**
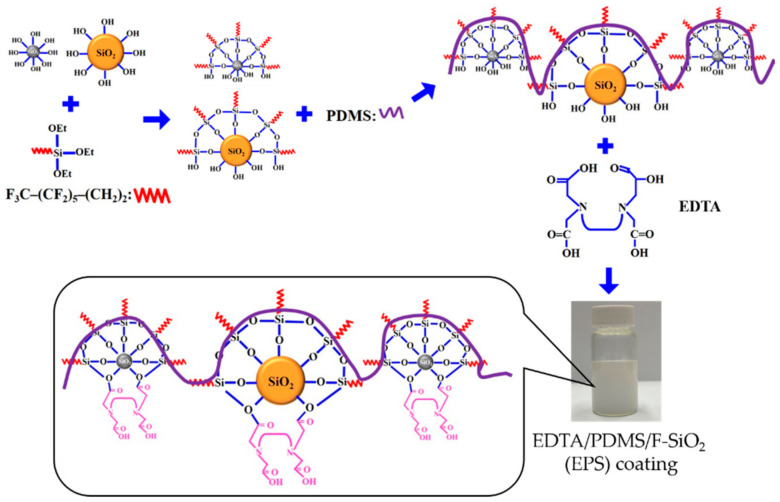
Schematic illustration of the EPS composite coating solution preparation.

Hydrophobic silica particles were prepared by hydrolysis and condensation of a FAS solution in EtOH, followed by the dispersion of PDMS into the solution. Subsequently, EDTA was added to the hydrophobic solution of F-SiO_2_ and FAS. EDTA readily cross-linked with a hydrophobic solution, and a suitable amount of free carboxylic acid groups in EDTA or free hydroxyl groups in the hydrophobic solution could bond with cotton fabric, resulting in a strong adhesion between the coating and substrate. This coating solution was then directly applied onto the fabrics using dip coating; however, other techniques, such as spray coating, could also be used for various substrates. The procedure for preparing the EPS composite coatings is shown in Figure 1. For comparison, we also prepared PDMS/F-SiO_2_ (PS), as described previously in the absence of EDTA.

### 2.3. Characterization

WCA and SA were measured with a contact angle meter (OCA15EC, DataPhysics, Filderstadt, Germany) using 10 μL droplets at room temperature. The surface morphology of the samples was observed by scanning electron microscope (SEM, Phenom Pro, Eindhoven, The Netherlands) at different magnifications. Prior to SEM measurements, a very thin Platinum (Pt) layer was sputter-coated on the sample to enhance its conductivity. Atomic force microscopy (AFM, CSPM-4000, California, USA) measurements were carried out to study the sample surface roughness profiles. Sandpaper abrasion tests were used to study the wear-resistance properties of the as-prepared surfaces. 

For the oil/water separation application, the EPS-coated textile was fixed well and the water/dichlorohexane mixtures with a volume ratio of 5:5 were poured on the coating. After each separation, the coating was dried in an oven and subsequently used for the oil/water separation. The light stability was characterized by exposing the coating to UV irradiation (18 W, 254 nm) from 1 to 7 days with a distance of 5 cm between the light source and the surface. Chemical stability was evaluated by measuring the WCA after the coating immersion into nitric acid (pH = 1) and sodium hydroxide (pH = 14) solution for 7 days. To evaluate the self-cleaning and antifouling performance, the coatings were immersed into a 10 ppm Congo red solution for 10 min. Sandpaper abrasion tests were used to study the wear resistance and surface robustness. The sample was placed face-down to sandpaper (standard sandpaper, grit no. 1200), moved for 10 cm along the ruler under a weight of 100 g, and then moved back along the same path. This process was defined as one abrasion cycle, wherein the surface was abraded longitudinally and transversely in each cycle. The WCAs after each abrasion cycle were measured.

## 3. Results and Discussion

### 3.1. Influence of Different-Sized SiO_2_ Proportion on Hydrophobicity

**Figure 2:**
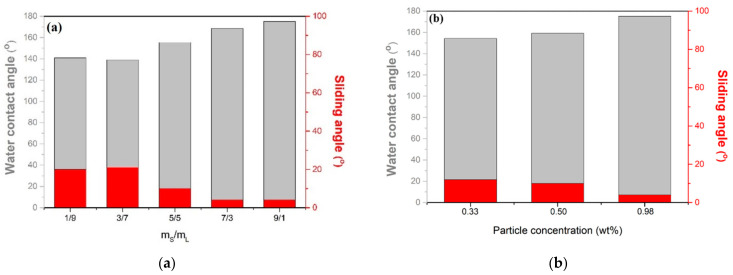
Influence of different-sized SiO_2_ particles proportion on the hydrophobicity of the coating. (**a**) The mass ratio of particles S and L in the coating and (**b**) the particle concentration of coatings.

The influence of different-sized particles proportion on the hydrophobicity of coatings is shown in Figure 2. The SiO_2_ particles with diameters of 12 nm and 10.5 µm are defined as particle S and particle L, respectively. The addition of S and L particles in the composite coating resulted in WCAs of 121° and 110°, respectively, indicating that the SH surface was not successfully prepared. As shown in Figure 2a, a WCA up to 139° was obtained after the addition of particle S. When the mass ratio of particle S/L was equal to 1(0.5/0.5), the WCA was 155.4° and reached the SH surface. Furthermore, when the mass ratio of particle S/L was 9, the WCAs could reach a maximum of 175.2°. These results demonstrate that with an increasing mass ratio of particle S/L, the micro/nanostructures are more obvious, and more air is trapped on the surface, which enhances the superhydrophobic property. The formation of micro/nanostructures by the proper combination of both S and L particles is one of the necessary conditions to prepare SH surfaces. Nevertheless, when the mass ratio of smaller/larger particles is less than 1, the larger particles per unit area will increase, which indicates that the lack of micro/nano rough structures on the surface could effectively trap a large fraction of air to create SH surfaces. 

The effect of particle concentration in the EPS composite paint on hydrophobicity was investigated. To obtain a transparent coating (transmittance > 90%), three EPS coatings with different lower concentrations were carried out, wherein the mass and S/L ratio of the particles were fixed at 9. Thus, the relationship between the particle concentration and WCAs was obtained, and it is shown in Figure 2b. The WCAs increased with increasing concentrations and reached 175.2° when the particle concentration was 0.98 wt%. However, the WCAs decreased with a further decrease in particle concentrations. These results demonstrate that with decreasing particle concentration, the micro/nanostructures are less obvious, and the roughness of the substrate surface cannot be effectively created; less air is trapped on the surface, which further leads to a decrease in WCAs.

### 3.2. Superhydrophobic Coatings for All Substrates

**Figure 3:**
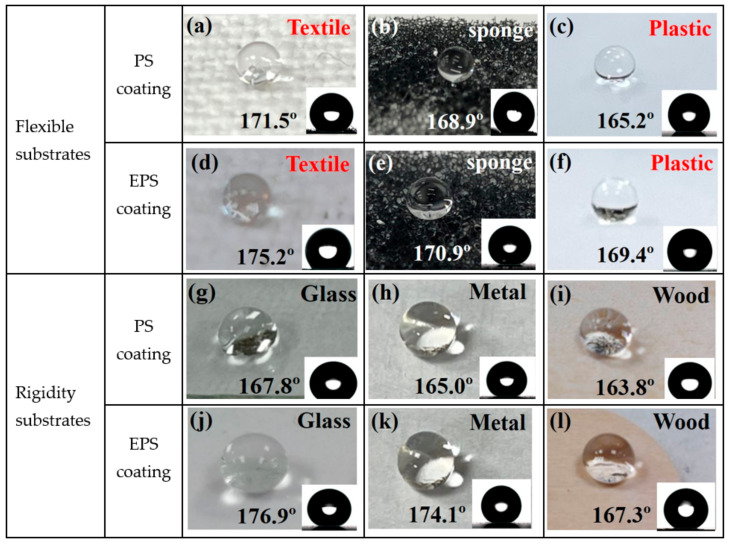
(**a**–**f**) Optical images of blue water droplets on flexible substrates coated with PS and EPS, such as textile, sponge, and plastic surfaces. (**g**–**l**) Optical images of water droplets on rigid substrates coated with PS and EPS, such as glass, wood, and metal substrates. Insets: Optical images and CAs of water droplets on the surface.

These excellent superhydrophobic coatings and the reliability of the obtained superhydrophobic surface are the results of producing lots of interspaces that trap air inside and form air cushions. The air cushions significantly increase the gas–liquid interfaces and effectively reduce the contact of the coating surface with the liquid in the wetting process. The substrates for superhydrophobic coatings are different depending on the applications. In this study, the prepared PS and EPS paints were coated on rigid substrates (such as glass, metal, and wood) and flexible substrates (such as textile, sponge, and plastic), respectively, as shown in Figure 3a–l. The droplets on all substrate surfaces were present in the form of complete water balls. The insets are the optical images of water droplets and measured WCAs. The results show that the prepared superhydrophobic paints are suitable for all rigid and flexible substrates to form the SH surface, and the WCA of EPS paint is slightly higher than PS paint.

### 3.3. Oil/Water Separation Properties

**Figure 4:**
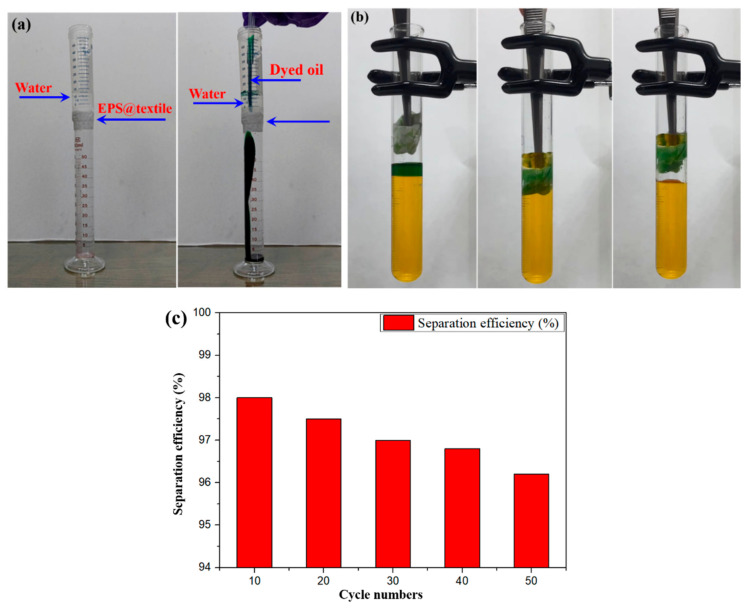
The optical photographs of oil/water separation using a fabric coated with EPS@textile as (**a**) filtering membrane and (**b**) adsorbent; (**c**) durability test of separation efficiency using EPS@textile.

The EPS paint coating onto the textile (EPS@textile) shows different wettability for oil and water. Herein, oil/water separation experiments using a self-made device were performed, as shown in Figure 4. The EPS@textile, as a filter membrane, was fixed between two vessels to separate the oil and water mixture. When water (originally in the upper vessel) and mineral oil (dyed with blue oil base ink) were poured onto the EPS@textile (Figure 4a), the dyed mineral oil spread out rapidly. It penetrated through the EPS@textile and dropped into the lower vessel, whereas the water remained in the top vessel due to the different wettability for oil and water (see Video S1, Supporting Materials). The oil/water mixed solutions were successfully separated using the superhydrophobic EPS@textile, demonstrating that the superhydrophobic fabric has high oil/water separation efficiency without the application of any external force.

In addition to separating oil/water mixed solutions, the EPS@textile could also treat non-water-soluble pollutants floating in the water. Blue oil-based ink was poured into the bottle, which contained water dyed with methyl orange to form a thin layer, then the oil/water mixture was stirred and allowed to stand for 5 min. As shown in Figure 4b, a piece of EPS@textile sank below the oil/water interface. It strongly repelled water and selectively adsorbed the oil when it touched the oil surface. After selectively adsorbing oil from the oil/water mixture, a fresh water surface was left. The EPS@textile had a 3D porous superhydrophobic structure, which could selectively adsorb toluene floating on the water surface. Compared with other complicated processes, the simplicity of the operations studied here makes this coating potentially useful for practical applications in our daily life.

Moreover, separation efficiency could be defined as the ratio of the difference in oil concentration between the original mixture and the collected solution to the original oil concentration [26,27]. Accordingly, the separation efficiency of the EPS@textile and PS@textile for the first separation was approximately 98.3% and 96.1%, respectively, indicating that the EPS@textile exhibited better separation performance than PS@textile. This result could be attributed to the EDTA treatment, which improved the superhydrophobicity of the EPS@textile. Next, a durability test of separation efficiency using EPS@textile was conducted. As shown in Figure 4c, as the cycle number of separation increased to 10, 20, 30, 40, and 50, the separation efficiency gradually decreased to 98.0%, 97.5%, 97.0%, 96.8%, and 96.2%, respectively. The separation efficiency after 50 separation cycles was still maintained higher than 96%, suggesting the good durability of the EPS@textile.

### 3.4. Chemical and UV Light Irradiation Stability

**Figure 5:**
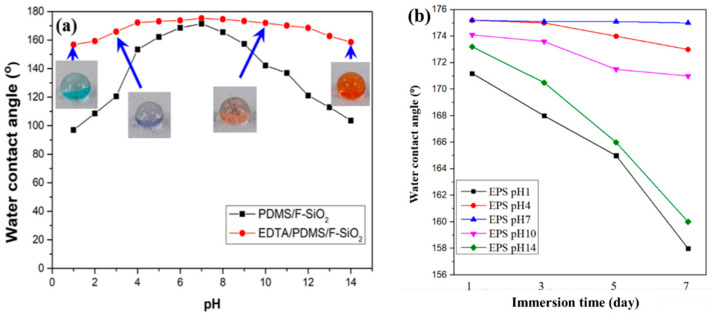

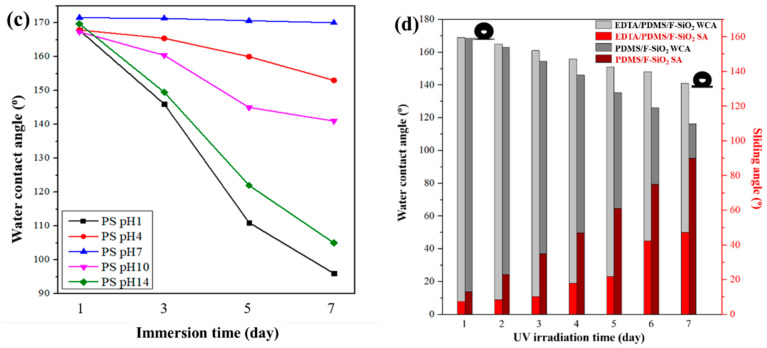
(**a**) Variations in WCAs of as-prepared superhydrophobic EPS@textile and PS@textile with pH. The down inset is the optical image of a water droplet with different pH values; the effect of pH value on WCAs of (**b**) EPS@textile and (**c**) PS@textile with the immersion time; (**d**) variations in WCAs and SAs of the EPS@textile with UV irradiation time. Insets are photographs of a water droplet on the EPS@textile after 1 and 7 days of UV irradiation.

In various applications of superhydrophobic coatings, particularly oil/water separation, the superhydrophobic coatings are usually exposed to harsh environmental conditions that are commonly encountered in practical settings. Thus, the acid and alkali resistance of the prepared EPS@textile and PS@textile were evaluated. The prepared coatings were immersed into aqueous solutions with pH values of 1 and 14 for a certain time. Their WCAs were recorded after removal from the solutions. As shown in Figure 5a, the WCAs of EPS@textile exhibited smaller variation with pH values of 1 and 14 than that of PS@textile, indicating that the prepared EPS@textile showed perfect acid and alkali corrosion resistance. Additionally, the WCAs of the EPS@textile were maintained higher than 158°, showing its high superhydrophobicity to a series of acidic and alkaline solutions. These results imply that EDTA improved acid/alkali corrosion resistance and significantly enhanced chemical durability. This can be attributed to the improved crosslinking of EDTA as a tetracarboxylic acid with both SiO_2_ and FAS when added together in one solution. This resulted in a densely rough coating and strong bonding with the textile surface, leading to improved stability and durability.

Furthermore, in order to evaluate the effect of pH value on coating durability, the WCAs of EPS@textile and PS@textile were measured with varying immersion time, as shown in Figure 5b,c, respectively. Minimal changes in WCAs were observed for both coatings (EPS and PS) when the textiles were immersed in a neutral state (pH = 7). However, in highly acidic (pH = 1) or alkaline (pH = 14) environments, the WCAs sharply decreased with increasing immersion time. The decline in WCAs was more significant in highly acidic environments (pH = 1) than in highly alkaline ones (pH = 14). In addition, the sequence of WCAs decline was pH 1, 14, 10, 4, and 7 for both EPS@textile and PS@textile. Additionally, the EPS samples exhibited a smaller decrease in contact angle compared with PS, indicating that EPS@textile had more stable coatings in solutions with different pH values than PS@textile.

On the other hand, the superhydrophobic EPS@textile coatings for oil/water separation are used outdoors. Therefore, UV light irradiation stability tests of the superhydrophobic coatings are necessary. To investigate the light irradiation stability, the prepared EPS@textile coatings were exposed to the irradiation of UV light (18 W, 254 nm) for 1 to 7 days, and the WCAs and sliding angle (SA) were also recorded, as shown in Figure 5d. As the exposure time increased, the WCAs slowly decreased, accompanied by an increase in SA, but still remained above 151° until the 5th day. However, as the exposure time was further prolonged to the 7th day, the WCAs continued to decrease slowly, and the coating gradually lost its superhydrophobicity (WCA of 143°). It could be inferred that UV light irradiation with a wavelength of 254 nm induced the decomposition of EDTA and its intermediate products on the textile surface, leading to the decrease in WCA and the increase in SA. Although these results agreed with the reported literature, the EPS@textile coatings retained their hydrophobicity even after 7 days of UV light irradiation, and the performance of EPS@textile was still superior to that of PS@textile.

### 3.5. Self-Cleaning and Antifouling Properties

**Figure 6:**
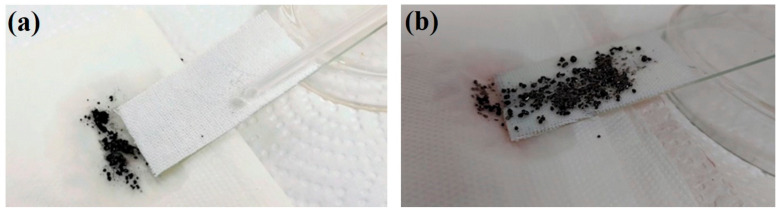
Self-cleaning property of the (**a**) EPS@textile and (**b**) bare textile against carbon black particles.

Water repellency implies the outstanding self-cleaning and antifouling properties of the superhydrophobic surfaces. The self-cleaning and antifouling functions of the prepared coating were tested to demonstrate the feasibility of the superhydrophobic and oleophobic coating in practical applications. The superhydrophobic EPS@textile was stained by carbon black (Cabot VULCAN^®^ XC-72R, Tuscola, IL, USA) particles. Different processes of the self-cleaning effect are shown in Figure 6. The fabric sample was bonded on a transparent glass slide surface with a slight slope. When water droplets were individually dropped on the uncoated substrate, water could not effectively remove the carbon black particles on the EPS@textile surface. As shown in Figure 6a, compared with the bare textile in Figure 6b, carbon black particles on the coated surface were wrapped in the liquid droplet and rolled from the EPS@textile spontaneously without leaving any residue, leading to a clean and dry EPS@textile surface. The EPS@textile exhibited an excellent self-cleaning performance, which can be attributed to water droplets rolling on the superhydrophobic surface (see Video S2, Supporting Materials).

**Figure 7:**
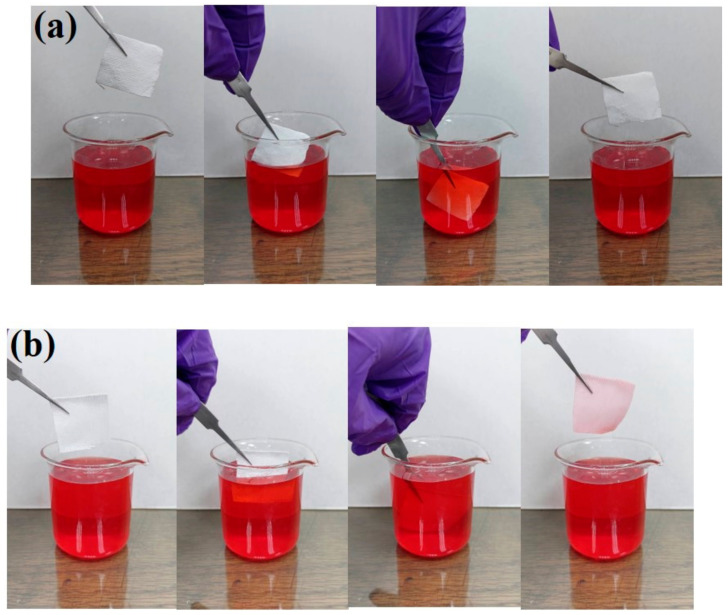
Antifouling tests of the (**a**) EPS@textile and (**b**) bare textile against water.

Furthermore, the antifouling performance of the coated fabric surface toward rhodamine B liquid pollutant was also studied. Both the EPS@textile and bare textile were immersed into a beaker containing colored liquid. The superhydrophobic fabric initially floated on the rhodamine B liquid due to the high water repellence and then immersed into the rhodamine B liquid just by using external forces. After immersion, the textile without and with the superhydrophobic EPS coatings was pulled out, as shown in Figure 7. The textile with the superhydrophobic coating remained very clean (Figure 7a); however, the textile without superhydrophobic coating was prominently contaminated by the rhodamine B liquid (Figure 7b). These results suggest that the prepared superhydrophobic EPS@textile has excellent self-cleaning and antifouling abilities. This was due to the superhydrophobic nature of the uncoated fabric and high hygroscopicity, while the trapped air between the coated superhydrophobic fabric and colored water hindered the liquid from penetrating the surface.

### 3.6. Wear-Resistance Test

**Figure 8:**
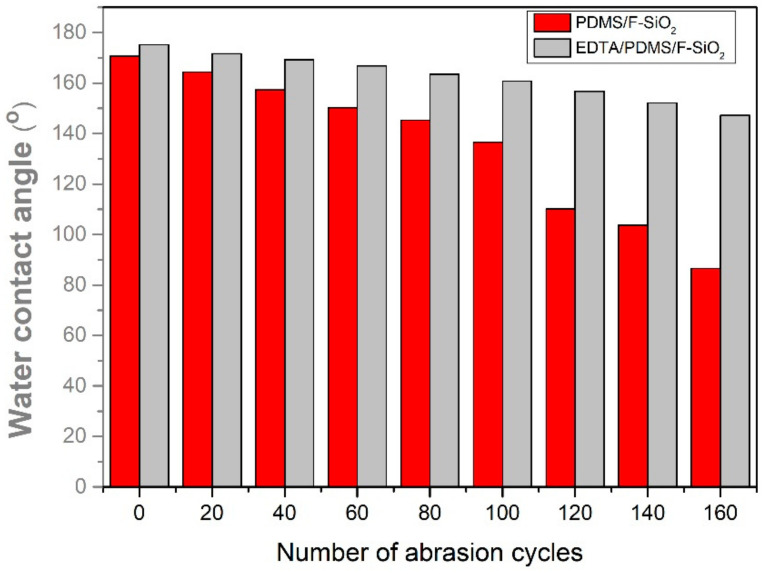
WCA variations in the EPS@textile and PS@textile after resistance test abrasion cycles.

The superhydrophobic micro/nano-scale binary rough structures, which were created by two different-sized SiO_2_ particles, were susceptible to external forces and damages. The mechanical stability of the artificial superhydrophobic coating is one of the most critical properties for practical applications. Therefore, we evaluated the coating–substrate adhesion force and mechanical robustness of the prepared superhydrophobic EPS@textile through a loaded sandpaper abrasion test. To explore the wear resistance of the EPS@textile, a weight of 100 g was fixed on top of the sample, and the uniform sliding abrasion was carried out on 1000-grit sandpaper at a speed of approximately 1.0 m/s. The pressure to bear was approximately 9.33 kPa. The EPS@textile remained hydrophobic at 147.2° after 160 abrasion cycles. This is mainly attributed to the improved crosslinking of EDTA with both F-SiO_2_ and FAS to create a robust micro/nanostructure and strong bonding on the cotton surface, which resulted in stability and durability, as shown in Figure 8. The comparison of PS@textile without EDTA can offer strong bonding with textiles. Thus, after 160 abrasion cycles, most of the matrix material is exposed, and hydrophobicity is lost, causing the WCA to rapidly reduce to approximately 80°.

## 4. Conclusions

In contrast to the previously reported complex processes for the preparation of durable and effective oil/water separation superhydrophobic coatings, a facile route to prepare mechanically robust transparent superhydrophobic coating was developed in this study by a simple and cost-effective dip coating or spraying method. Here, an EDTA/PDMS/F-SiO_2_ hierarchical structure was constructed by fluorinated micro/nanoparticles, a binder of silicone resin, and a crosslinking agent. The prepared SH coating exhibits excellent water-repellency performance with a WCA greater than 175° and an SA less than 10°. Additionally, the coating shows excellent durability and remarkable superhydrophobicity for oil–water separation, abrasion resistance, UV light irradiation stability, chemical stability, self-cleaning, and antifouling under various harsh conditions. Therefore, for the preparation of robust transparent superhydrophobic coatings, the current work will provide new insights and valuable information for future studies and practical and large-area applications.

## Data Availability

Not applicable.

## Supplementary Materials

The following supporting information can be downloaded at: https://www.mdpi.com/article/10.3390/membranes13040401/s1, Video S1: presented that the dyed mineral oil penetrated through the EPS@textile and dropped into the lower vessel, whereas the water remained in the top vessel; Video S2: presented that the EPS@textile exhibited an excellent self-cleaning performance, which can be attributed to water droplets rolling on the superhydrophobic surface.
